# Supplementary material for: Nettle, a Long-Known Fiber Plant with New Perspectives
Source: Materials (Basel). 2022 Jun 17;15(12):4288. doi: 10.3390/ma15124288 (PMC9230748; doi:10.3390/ma15124288)
Supplement: Supplementary file 1 [file materials-15-04288-s001.zip › materials-1731520-supplementary.pdf]

Nettle, a long-known fiber plant with new perspectives: a review

Chloé Viotti[1], Katharina Albrecht[2], Stefano Amaducci[3], Paul Bardos[4], Coralie Bertheau[1], Damien Blaudez[5], Lea Bothe[2], David Cazaux[6], Andrea Ferrarini[3], Jason Govilas[7], Hans-Jörg Gusovius[8], Thomas Jeannin[7], Carsten Lühr[8], Jörg Müssig[2], Marcello Pilla[3], Vincent Placet[7], Markus Puschenreiter[9], Alice Tognacchini[9], Loïc Yung[5] & Michel Chalot[1,10*],

[1]UMR Chrono-environnement, CNRS 6249 - Université de Bourgogne-Franche-Comté, F-25000 Besançon, France.

[2] The Biological Materials Group, Dept. Biomimetics, HSB – City University of Applied Sciences Bremen, Neustadtswall 30, D-28199 Bremen, Germany.

[3]Department of Sustainable Crop Production, Università Cattolica del Sacro Cuore, Via Emilia Parmense 84, 29122, Piacenza, Italy.

[4]r3 Environmental Technology Ltd, Earley Gate, Reading, RG6 6AT, United Kingdom

[5]Université de Lorraine, CNRS, LIEC, F-54000 Nancy, France.

[6]Inovyn, Tavaux, France.

[7]FEMTO-ST Institute, Department of Applied Mechanics – Université Bourgogne Franche-Comté, Besançon, France.

[8]Leibniz Institute for Agricultural Engineering and Bioeconomy (ATB), Max-Eyth-Allee 100, 14469 Potsdam, Germany.

[9]University of Natural Resources and Life Sciences Vienna, Vienna, Austria

[10]Université de Lorraine, Faculté des Sciences et Technologies, F-54000 Nancy, France.

* Correspondence to [michel.chalot@univ-fcomte.fr]
Université de Bourgogne Franche-Comté
UMR 6249 Laboratoire Chrono-environnement
Pôle Universitaire du Pays de Montbéliard
4 place Tharradin,
25 211 MONTBELIARD

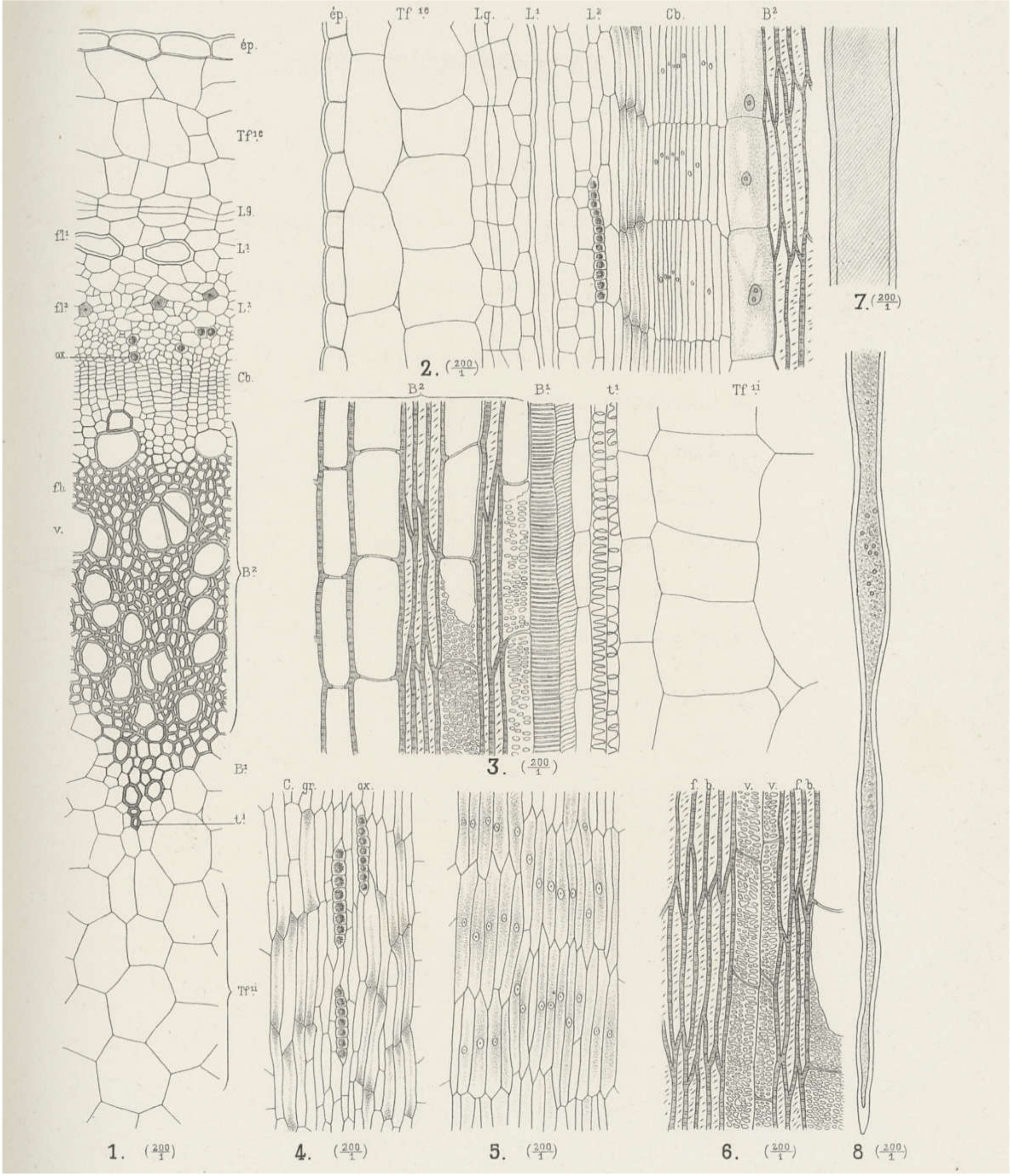

Figure S1. Histology plate of the nettle stem from Gravis (Gravis 1885). 1. Portion of a cross-section showing the primary and secondary productions of the vascular bundle. 2. Radial section passing through the middle of a vascular bundle (from epidermis to secondary xylem). 3. Continuation of figure 2 (from secondary xylem to pith). 4. Tangential section passing through the secondary phloem. 5. Tangential section passing through the cambium. 6. Tangential section passing through the secondary xylem. 7. Primary bast fiber – middle region. 8. Primary bast fiber – extremity of the same fiber. Tf^{1i} primary fundamental tissue - internal, Tf^{1e} primary fundamental tissue - external, B^{1} primary xylem, B^{2} secondary xylem, t^{1} first trachea, v. vessel, f.b. xylem fiber, Cb. cambium, ox oxalate of lime, fl^{1} primary bast fiber, fl^{2} secondary bast fiber, L^{1} primary phloem, L^{2} secondary xylem, Lg cork (phellem), C.gr. barred cells, ep. Epiclethra.

Table S1. Macro (g/kg dry weight) and trace (mg/kg dry weight) elements concentrations reported in the different tissues of Urtica dioica from uncontaminated sites.

Author	(Đurović et al. 2017)	(Tack & Verloo 1996)	(Kara 2009)	(Rafajlovska et al. 2013)		(Kabata-Pendias 2011)			
Origin	Serbia,	Belgium,	herbal infusion	Macedonia	Macedonia	Approximate Concentrations of Trace Elements in Mature Leaf Tissue Generalized for			
Plant part	Leaf	Whole plant	Leaf	Leaf	Stems	Leaf	Leaf	Leaf	Leaf
	mean ± SD	mean ± IQR	mean ± RSD %	range	range	deficient	sufficient/ normal	excessive/ toxic	Tolerable in agronomic crops
Macro-elements									
K	33.9 ± 4.08		17.5 ± 5.7						
Mg	8.69 ± 0.31		7.32 ± 5.3	25.1 -35.6	7.9 - 16.7				
Ca	28.6 ± 0.825		38.4 ± 7.0	26.3 - 50.1	8.9 - 14.2				
P			3.36 ± 7.5						
Na	0.296 ± 0.013		0.128 ± 7.8						
Trace elements									
Cu	8.00 ± 0.38	14 ± 5	11.2 ± 3.5	11.1 - 17.5	7.9 - 15.3	2-5	5-30	20-100	5-20
Mn	81.40 ± 4.24	291 ± 258	66.5 ± 1.7	4.03 - 20.8	3.3 - 19.1	10-30	30-300	400-1000	300
Zn	18.03 ± 0.60	113 ± 82	22.0 ± 4.9	17.0 - 27.4	15.3 - 25.9	10-20	27-150	100-400	50-100
Cr	0.31 ± 0.03	6.6 ± 2.7	1.77 ± 5.4				0.1-0.5	5-30	2
Sn	0.49 ± 0.05								
Ni	0.03 ± 0.01	9.1 ± 2.9	2.0 ± 3.5				0.1-5	10-100	1-10
Co		0.8 ± 0.4	0.50 ± 4.7	0.11 - 0.21	0.10 - 0.18		0.02-1	15-50	5
Sr			134 ± 4.0						
Ba			37.5 ± 4.6					500	
Fe	151 ± 5.41	432 ± 216	999 ± 6.8						
Pb	0.18 ± 0.02	34 ± 19					5-10	30-300	0.5-10
Cd	0.02 ± 0.01	0.43 ± 0.2					0.0.-0.2	5-30	0.05-0.5
Hg	ND							1-3	0.2
As	0.35 ± 0.26						1-1.7	5-20	0.2

Table S2. Selected initiatives on nettle cultivation and selection since the late 1990s.

Project	Source of funding	Application	Description
From nettle to textile I (1997-2000) et II (2001-2003)	Agricultural Research Centre of Finland and College/Crafts and Design department	Cultivation, fiber, textile	Development of fiber cultivation and treatment methods (biotechnological retting and mechanical treatment) for the textile industry
Nettle – reintroduction of stinging nettle cultivation as a sustainable raw material for the production of fibers and cellulose. FAIR-ST-8356 et FAIR-CT98-9615 (1999-2001)	EU-FP4_FAIR	Cultivation, fiber, textile	Development of organic farming methods, testing of different fiber processing methods, and textile manufacturing
Natural textiles made of nettle – innovative technology and product development for the textile industry (1999-2002)	Institute of Plant Production and Breeding, University of Göttingen; Thüringisches Institut für Textil – und Kunststofforschung e.V .; Institute of Applied Botany, University of Hamburg; Spremberger Tuch GmbH; Langhein-Textil GbR (Germany)	Cultivation, textile	Clothing production. Development from cultivation methods in organic agriculture to manufacturing
STING (2004-2008) « Sustainable Technology In Nettle Growing »	Department for Environmental Food and Rural Affairs, UK	Fiber, physiology and phytochemistry	Optimization of fiber extraction and production. Comparing wild nettle plants in the UK with clones selected for their high fiber content
ICCOG (2008-2009) « Identification and characterization of some clones of nettle and Spanish broom for textile and phytotherapic use »	Tuscany region Italia	Textile, medicine, cosmetic	Identify and characterize clones with a high amount of fiber and metabolites for the cosmetic and phytotherapeutic sectors, as well as antifungal and antibacterial properties
PRIN 2009 « Medicinal and dyeing-plants natural extracts: characterization, and innovative poly-use	Italian Ministry of Research	Biocide	Evaluation of different aqueous nettle extraction methods on natural antioxidant content and

of nettle, daphne, lavender and chestnut tannins » (2010-2012)			effects on aphids, antimicrobial and antifungal properties
Plant resources for food and non-food use in Tuscany Region (2010-2011)	Tuscany region Italia	Fiber	Characterization and life cycle assessment of several nettle products
Study of alternative fiber plants in Lithuania (2010-2013)	Part of the LRCAF program "Biopotential and Quality of Plants for Multifunctional Use" Lithuania	Cultivation	Crop yield, crop density, morphological indices, recommendations to producers, and possible valorizations
LORVER (2013-2018)	Region Lorraine<br>FEDER<br>France	Fiber	Creating a non-food plant biomass production chain by developing degraded sites and using industrial by-products in Lorraine
NEWFIBER (2015-2018)	Region Lorraine<br>FEDER<br>France	Fiber, textile	Development of an environment-friendly defibration process and fiber for the textile industry
PHYTOFIBER (2017-2020)	ADEME, France	Fiber	Valorize plant fibers from biomass derived from contaminated soils through the production of manufactured products
CABERNET (2017-2020)	Fonds National de la Recherche Luxembourg	Fiber	Study of molecular mechanisms governing fiber formation
ORTIKA (2018-2019)	H2020	Fiber and textile	Crop production with a next clone variety, optimization of the machine for fiber extraction process, and implementation of a natural fixative method
NETFIB (2019-2022)	SusCrop – ERA NET	Fiber	Crop production on marginal lands
ARKNOKK (2020-2023)	FEDER, Finland	Cultivation and valorization	Development of traditional organic cultivation methods for the cultivation of nettle to be used as raw material for existing products and new products to be developed.

Table S3. Trace elements concentrations (mg / kg dry weight) reported for Urtica dioica L. tissues across a range of studies. Data reported are means. Plant tissues : R, roots; S, shoots; S, stems; F, fibers; I, inflorescence. Growth condition : 1. US, soil with lead-arsenate used as pesticide, pot experiment; 2. Bangladesh, pot experiment; 3. Perlite with $CdSO_4$ and $ZnSO_4$ applied in solution, pot experiment ; 4. France, nettle sampled in situ at contaminated sites 5. Poland, nettle sampled in situ; 6. Ohio, nettle sampled in situ; 7. Turkey, nettle sampled *in situ*.

Author	(Codling & Rutto 2014)		(Shams et al. 2010)			(Sinnett et al. 2009)	(Jeannin et al. 2020)			(Paukszto & Mirosławski 2019)				(Spongberg et al. 2008)			(Güleryüz et al. 2008)		
Growth condition	1.		2.			3.	4.			5.				6.			7.		
Plant tissues	R	S	R	S	L	L	S	F	L	R	S	L	I	R	S	L	R	S	L
Cu							3.3	2.9	9.4					241.6-562.8	34.0-60.2	26.9-138.3	19-146	15-52	71-127
Mn										25-30	17-21	26-46	30-33	36.3-179.3	0-37.4	39.7-86.5			
Zn							24.0	47.5	31.9					195.5-458.4	48.5-73.2	45.5-130.1	45-80	26-46	97-280
Cr			12-20	1	5-10									24.3-37.8	17.1-22.5	14.4-21.6	12-64	5-18	10-32
Pb	83.3-275.0	0.8-4.4					1.9	1.3	2.0										
Cd						0.1-24.9	1.0	1.1	0.9										
Hg							0.01		0.03										
As	100.0-653.3	33.3-123.3																	
